# Live longer, retire later? Developments of healthy life expectancies and working life expectancies between age 50–59 and age 60–69 in Europe

**DOI:** 10.1007/s10433-020-00592-5

**Published:** 2020-12-03

**Authors:** Daniela Weber, Elke Loichinger

**Affiliations:** 1 Wittgenstein Centre for Demography and Global Human Capital (IIASA, VID/OEAW, WU), International Institute for Applied Systems Analysis (IIASA), 2361 Laxenburg, Austria; 2 Health Economics and Policy Division, Vienna University of Economics and Business, Welthandelsplatz 1, 1020 Vienna, Austria; 3 Federal Institute for Population Research, 65185 Wiesbaden, Germany

**Keywords:** Economic activity, Retirement, Cognition, Physical health, Old age, SHARE

## Abstract

Europe’s population is ageing. Statutory retirement ages are commonly raised to account for continuous increases in life expectancy. In order to estimate the potential to increase statutory and consequently effective retirement ages further, in this study, we investigate the relationship between partial working life expectancy (WLE) and three health expectancies that represent health aspects important for work ability and employability between ages 50 and 59 as well as 60 and 69 for women and men in Europe. We also explore the association between these four indicators and the highest level of educational attainment. We apply Sullivan’s method to estimate WLE and three selected measures that capture general, physical, and cognitive health status of older adults for 26 European countries since 2004. Over time, WLEs increased significantly in the younger age group for women and in the older age group for both sexes. The expected number of years in good physical health have continuously been higher than any of the other three indicators, while the expected number of years in good cognitive health have shown a noticeable increase over time. The investigation of the relationship between education and each life expectancy confirms the well-established positive correlation between education and economic activity as well as good health. Our results indicate potential to extend working lives beyond current levels. However, significant differences in the expected number of years in good health between persons with different levels of education require policies that account for this heterogeneity.

**Electronic supplementary material:**

The online version of this article (10.1007/s10433-020-00592-5) contains supplementary material, which is available to authorized users.

## Introduction

Life expectancies at birth as well as at adult ages have been increasing in Europe for decades. This trend in combination with the in many countries persistently low levels of fertility entails a shift in populations’ age compositions towards older age groups. This development is prone to endanger financial sustainability in several areas of social security systems, one of them being pension systems, as many European countries provide public pensions on a pay-as-you-go basis. Further reforms to national pension systems are necessary to adjust social security systems to ageing populations (European Commission 2018).The timing when people leave the labour force and measures to support longer working lives are widely discussed at the country as well as the European Union level (Anderson et al. 2019; European Commission 2019), with workers’ health being an important factor (Eurofound 2019). Many countries have initiated and passed policy changes to gradually increase the official retirement age and general retirement ages are now above age 65 in several countries (see Tables 1 and 2). Consequently, labour force participation rates of persons 50 + have been increasing and are expected to continue to do so. One pertinent question in this context is how large the potential to increase working lives further is, given people’s health status. Or, put differently: what is the health status of persons at retirement ages and how does it compare to present lengths of working lives?

**Table 1:** Descriptive statistics for men (weighted with sample weights) from SHARE wave 7 in 2017 (sample size, share of men without activity limitations, good physical health, and good cognition ages 50 years and older) and life expectancy at age 60 in 2017, LFP rates for age group 60–64 in 2017, and the current general retirement age in 2019

Country	N (SHARE)	Without activity limitations 50 + (%)	Good physical health 50 + (%)	Good cognition 50 + (%)	Life expectancy at age 60	LFP rates 60–64	General retirement age (2019)
Austria	1392	52.0	76.5	82.2	22.5	39.8	65 yrs
Belgium	2261	53.4	78.2	77.3	22.4	34.9	65 yrs
Bulgaria	835	61.3	49.2	55.6	17.2	56.3	66 yrs 4 months
Croatia	1114	50.2	78.9	63.9	19	37.5	65 yrs
Cyprus	494	76.7	67.0	50.9	22.6	60.7	65 yrs
Czechia	1826	51.4	81.8	75.9	19.8	58.5	63 yrs 6 months
Denmark	1557	62.2	86.1	73.4	22.1	65.3	65 yrs 6 months*
Estonia	2166	44.5	78.0	63.0	18.8	60.4	63 yrs 6–9 months
Finland	920	55.5	81.5	69.0	22.3	53.2	65 yrs*
France	1486	58.2	77.8	70.8	23.4	31.2	66 yrs 2 months
Germany	1863	44.8	80.5	75.4	21.9	66.6	65 yrs 7 months
Greece	1431	77.5	62.6	68.2	22.4	42.9	67 yrs
Hungary	771	56.4	68.8	64.8	17.5	51.4	64 yrs
Italy	2163	67.5	67.3	59.7	23.3	52.1	66 yrs 7 months
Latvia	633	55.6	77.9	61.6	17	55.4	63 yrs 6 months
Lithuania	720	54.6	76.3	53.4	17.5	62.2	63 yrs 10 months
Luxembourg	589	54.6	79.1	71.2	22.6	21.3	65 yrs
Malta	550	73.3	52.8	55.9	23.1	43.8	63 yrs
Poland	2150	46.9	71.4	52.9	19.2	48.4	65 yrs
Portugal	233	39.4	53.0	44.0	22.1	56.2	66 yrs 5 months
Romania	894	55.2	64.7	48.9	17.8	42.2	65 yrs
Slovakia	938	71.2	65.2	62.0	18.6	37.4	62 yrs 6 months
Slovenia	1644	52.8	81.7	68.0	21.3	27.3	65 yrs
Spain	2307	63.6	58.1	53.6	23.3	51.2	65 yrs 6 months
Sweden	1553	62.5	81.2	71.2	23.4	74.6	65 yrs*
Switzerland	1133	70.1	79.1	78.9	24.1	71.7	65 yrs

^*^Represents the retirement age of the national pension, not the earnings-related pension

Sources: SHARE, Eurostat; for retirement ages: https://www.etk.fi/en/the-pension-system/international-comparison/retirement-ages/

**Table 2:** Descriptive statistics for women (weighted with sample weights) from SHARE wave 7 in 2017 (sample size, share of men without activity limitations, good physical health, and good cognition ages 50 years and older) and life expectancy at age 60 in 2017, LFP rates for age group 60–64 in 2017, and the current general retirement age in 2019

Country	N (SHARE)	Without activity limitations 50 + (%)	Good cognition 50 + (%)	Good physical health 50 + (%)	Life expectancy at age 60	LFP rates 60–64	General retirement age (2019)
Austria	1974	45.9	82.2	82.4	25.8	17.3	60 yrs
Belgium	2797	46.7	81.3	75.8	26.1	23.9	65 yrs
Bulgaria	1121	51.8	64.9	50.9	21.8	39.4	66 yrs 4 months
Croatia	1366	41.6	79.1	60.7	23.2	20.6	62 yrs
Cyprus	709	68.9	57.7	53.4	25.7	37.1	65 yrs
Czechia	2659	42.5	86.3	79.8	24	30.3	63 yrs 2 months
Denmark	1823	55.1	86.8	81.3	25.1	52.3	65 yrs 6 months*
Estonia	3280	38.3	81.4	73.8	25	60.3	63 yrs 6–9 months
Finland	1063	47.6	83.2	77.3	26.5	54.3	65 yrs*
France	2003	52.9	80.4	73.4	28	31.7	66 yrs 2 months
Germany	2060	40.0	84.9	80.6	25.5	55.3	65 yrs 7 months
Greece	1880	71.5	73.6	65.1	25.8	23.5	67 yrs
Hungary	1060	44.2	69.2	60.1	22.3	24.8	64 yrs
Italy	2603	58.4	76.3	57.5	26.9	31.7	66 yrs 7 months
Latvia	1083	45.1	83.5	65.1	23	53.5	63 yrs 6 months
Lithuania	1269	45.8	80.4	56.7	23.6	53.8	62 yrs 8 months
Luxembourg	686	46.8	76.1	72.3	26.1	14.2	65 yrs
Malta	696	63.9	65.4	57.7	26.6	18.6	63 yrs
Poland	2614	40.2	78.4	58.7	24.3	22.1	60 yrs
Portugal	281	39.5	70.8	32.6	26.6	40.3	66 yrs 5 months
Romania	1162	41.1	65.5	43.8	22.3	22	61 yrs 0–2 months
Slovakia	1052	57.4	73.3	59.9	23.3	28.5	62 yrs 6 months
Slovenia	2228	48.3	82.6	70.4	25.9	16.4	65 yrs
Spain	2801	56.7	65.1	53.0	27.8	38.5	65 yrs 6 months
Sweden	1781	51.3	84.6	77.1	25.9	69.2	65 yrs*
Switzerland	1339	61.5	82.6	81.8	27.3	54.6	64 yrs

^*^Represents the retirement age of the national pension, not the earnings-related pension

Sources: SHARE, Eurostat; for retirement ages: https://www.etk.fi/en/the-pension-system/international-comparison/retirement-ages/

On the micro-level, a lot of research exists that looks at the relationship between retirement and individual health, and depending on the model specification, the direction of effects and their magnitude vary widely. Boissonneault (2018) provides an in-depth overview of the impact of various aspects of health on retirement. Macro-level studies that investigate the relationship between economic activity and health are rare, in spite of their importance to understand developments on the population level (De Souza et al. 2019; Loichinger and Weber 2020). In this study, we take a macro-level approach in order to analyse the relationship between working life expectancy (WLE) and health status, focusing on three selected health dimensions that cover a range of health outcomes: general health, physical strength, and cognitive abilities. Our analysis builds on previous analyses where we presented developments of WLE over time by sex across Europe and presented comparisons of WLE and healthy life expectancy (HLE) at age 50 and WLE by education at age 50 for the year 2009 (Loichinger and Weber 2016). In the current study, we update our previous analyses and expand them in three crucial ways. First, we expand the analysis by two objective health outcomes and their development over time. Second, we analyse heterogeneity in WLE and all three health outcomes by education. Third, while we previously looked at the situation at age 50, we now focus on partial life expectancies between age 50–59 and 60–69, respectively. Focusing on these two age intervals provides a better understanding and allows for a more nuanced picture of developments right before and around current statutory old-age retirement ages.

An increasing number of studies analyse the development of working life expectancies in selected European countries (Dudel et al. 2018; Lorenti et al. 2019; Lozano and Rentería 2019; Pedersen et al. 2020; Robroek et al. 2020), with several of them including information on certain health aspects like disability, depressive symptoms or self-perceived health (de Wind et al. 2018; Lievre et al. 2007; Pedersen et al. 2019; van der Noordt et al. 2019; Wubulihasimu et al. 2015). The choice of health dimensions in our investigation is based on their relevance for individuals’ performance in the labour market. The ageing processes in muscle strength and cognition may negatively affect work ability, productivity, and employability (Tracy and Enoka 2002; Ypsilanti and Vivas 2011). When it comes to physical ability, adequate muscle power has been shown to be a prerequisite for optimal productivity (Shephard 2000) while decreased muscle strength is a predictor of physical limitations (Magee 2013). Hand-grip strength is a good indicator of muscle strength; it peaks at about ages 30 to 40 and decreases thereafter (Dodds et al. 2014; Steiber 2015). We use this measure to estimate physical ability. When it comes to cognition, studies have found that cognitive ability levels predict individual productivity better than any other observable individual characteristics and that they are increasingly relevant for labour market performance (Schmidt and Hunter 2004; Spitz-Oener 2006). Age-related cognitive decline is usually characterized by having difficulty recalling facts (Ritchie and Tuokko 2010), but may additionally affect processing speed (Salthouse 2010). Immediate word recall, our measure for cognitive health status, is a by now well-established indicator for cognitive functioning and abilities (Salthouse 2010). General health and performance in the labour market are investigated by a vast literature; overall poor health was identified to reduce the capability to work (García-Gómez et al. 2011; Lindeboom 2012) and moreover disabilities and health problems have been shown to increase the risk to leave the labour market (Wubulihasimu et al. 2015).

The analyses of the development of four life expectancy indicators—the aforementioned two labour market-specific health indicators, life expectancy in good general health and working life expectancy—across time and by gender for selected European countries as well as differences in these measures by education are the focus of this article. We start by comparing the four indicators for men and women for two advanced age groups in 2017. In addition to the subsequent visual presentation of trajectories between 2004 and 2017, we analyse the correlation between the four indicators. In the next step, we investigate the education-specific life expectancies of the four indicators in order to detect differences between socioeconomics subgroups of the population. Socio-economic status and health status are strongly correlated, and we expect to find significant heterogeneity between education groups when it comes to health and their ability to work beyond currently observed labour market exit ages.

## Methods

### Data sources

Labour force participation (LFP) rates by age (age groups 50–54, 55–59, 60–64, 65–69), sex and education were obtained from Eurostat and are based on the European Labour Force Survey (EU-LFS) (European Commission 2020). The LFP rate represents the share of the economically active population in each age group. Economically active persons comprise the employed and the unemployed, in line with the definition of the labour force by the ILO. In 15 out of the 26 European countries in our study, more than 50% of men age 60–64 are in the labour force. Among women, this applies to 8 out of 26 countries (see Tables 1 and 2). Period life tables for ages 0–85 + by sex and country were also provided by Eurostat (European Commission 2020). Further, education-specific life tables were calculated using sex-specific life expectancy information at birth by educational attainment level from Eurostat in 2016 for Denmark and Estonia and in 2017 for the thirteen further countries with available data (European Commission 2020). We distinguish three education categories following internationally comparable ISCED coding: at most lower secondary education (ISCED 0–2), upper secondary and post-secondary non-tertiary education (ISCED 3–4), and short-cycle tertiary education or higher (ISCED 5–8).

Individual health capacities related to work ability are represented by good general health (i.e. the absence of a severe long-term activity limitation) (Ribeiro et al. 2019), good physical functioning (i.e. good hand-grip strength) (Rentzsch et al. 2015), and good cognitive functioning (i.e. good episodic memory) (Ihle et al. 2015; Schmidt and Hunter 2004). We calculated prevalence rates of good general health as well as good physical and cognitive functioning with Survey of Health, Ageing and Retirement in Europe (SHARE) data from wave 1 (2004) to wave 7 (2017) (Börsch-Supan et al. 2013) including provided cross-sectional sampling weights. Details on the sampling strategy can be found elsewhere (Bergmann et al. 2019; Börsch-Supan and Jürges 2005). Good general health is measured by using the General Activity Limitation Indicator (GALI) (EHLEIS team 2011; Van Oyen et al. 2018). This indicator is widely used to monitor health developments within the European Union (Bogaert et al. 2018). Participants were asked to what extend they have been limited in activities they usually do because of a health problem within the past six months. Those who reported no activity limitations at all were categorized as having good general health. Hand-grip strength is an established indicator for morbidity and mortality (Bohannon et al. 2006; Massy-Westropp et al. 2011). This measurement (in kilograms) was taken twice per hand using a dynamometer with the maximum of the four measures considered. We distinguish those with good physical functioning as having grip-strength above 37 kg for men and 21 kg for women (Sallinen et al. 2010). Episodic memory is very closely linked with working memory, therefore we employ a word recall test in order to estimate our indicator for cognitive abilities. Participants had to immediately recall as many words as possible from 10 read out nouns within one minute, whereat a recall of at least five words out of ten is indicating good episodic memory (Skirbekk et al. 2012). Overall, the share of the population 50 + in good health is highest for physical functioning and lowest when looking at the share with no activity limitations (see Tables 1 and 2).

### Statistical analysis

We estimate the number of years a person is expected to be economically active and generally, physically, and cognitively healthy—called working, healthy, physical, and cognitive life expectancy (WLE, HLE, PLE, CLE, respectively)—by applying Sullivan’s method, following a standard approach to compare estimates of life expectancy indicators over time and across countries (Hytti and Valaste 2009; Jagger et al. 2011; Oyen et al. 2013). Prevalence rates are combined with life tables by survey year, sex, and country (Hytti and Valaste 2009; Sullivan 1971), resulting in estimates for the expected remaining years in a certain condition given the period values for survival and labour market participation and health status, respectively (Leinonen et al. 2015).

First, we investigate the number of years a person is expected to be without activity limitations or economically, physically or cognitively active in 2017. Second, trends in all indicators between 2004 and 2017 are investigated for these countries. We present results whenever available for 26 countries (i.e. Austria, Belgium, Bulgaria, Croatia, Cyprus, Czech Republic, Denmark, Estonia, Finland, France, Germany, Greece, Hungary, Italy, Latvia, Lithuania, Luxembourg, Malta, Poland, Portugal, Romania, Slovakia, Slovenia, Spain, Sweden, Switzerland) that participated at least in the last available wave of SHARE in 2017. Third, we analyse education-specific patterns of the four life expectancies for 15 European countries that education-specific life expectancies are available for (i.e. Bulgaria, Croatia, Czech Republic, Denmark, Estonia, Finland, Greece, Hungary, Italy, Poland, Portugal, Romania, Slovakia, Slovenia, Sweden). We calculate education-specific life tables using Brass relational model, combining life table information and education-specific life expectancy data (Preston et al. 2001). Our descriptive investigations uncover variations between education-specific partial life expectancies and the overall life expectancies. Uncertainty in four life expectancies due to usage of survey data is exemplarily provided by confidence intervals for all four indicators in 2015, where we had also most recent information on the sample size of sex-specific age groups of the EU LFS data (see Figs. 9, 10, 11 and 12 in Appendix). Further, we present the 95%-confidence intervals for the three health life expectancies by education in 2017 in the supplement.

In this study, we calculate partial working, healthy, physical, and cognitive life expectancies from ages 50 to 59 and ages 60 to 69 (Hickman and Estell 1969). Partial life expectancies allow comparing the number of years lived between the 50th and 60th birthday and 60th and 70th birthday, respectively. This concentration on the two decades covering late adult working life and the onset of retirement accounts for the fact that economic activity is very low after age 70.

## Results

### Differences in life expectancies across Europe in 2017

**Fig. 1:**
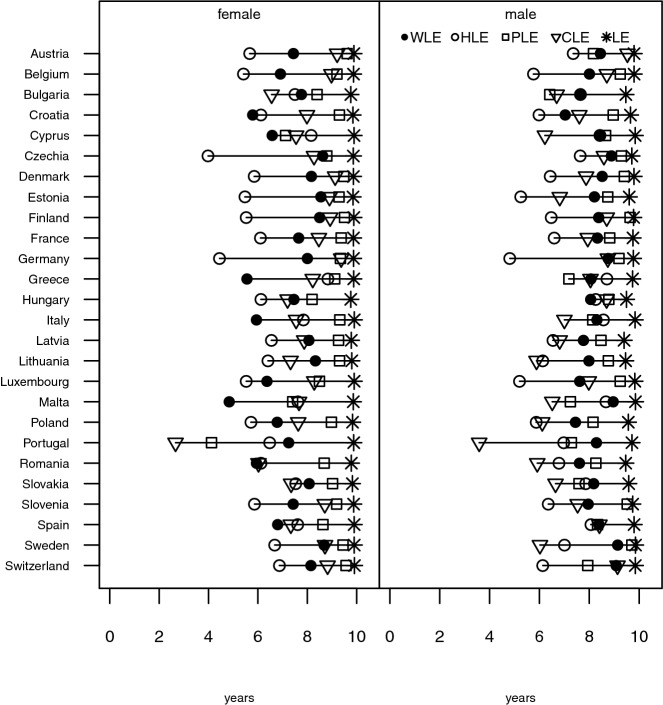
Partial life expectancies (WLE, HLE, PLE, CLE, and LE) between ages 50 and 59 for women and men for selected European countries in 2017

**Fig. 2:**
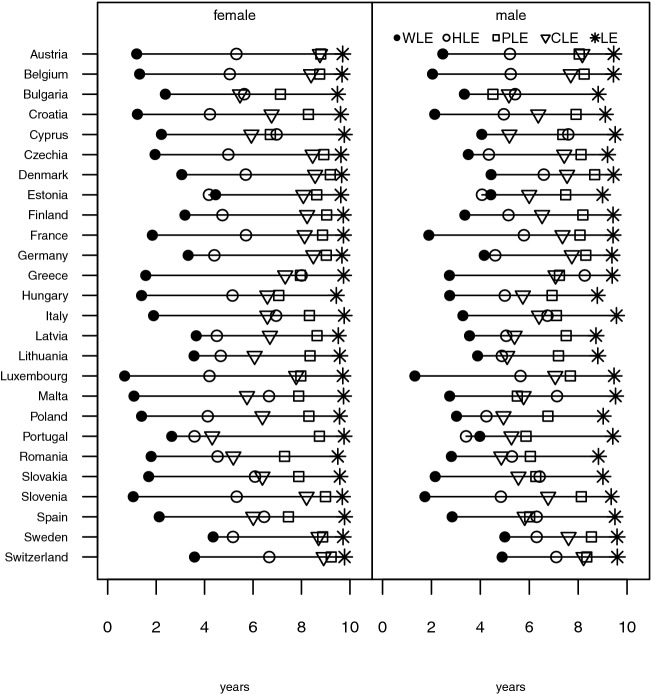
Partial life expectancies (WLE, HLE, PLE, CLE, and LE) between ages 60 and 69 for women and men for selected European countries in 2017

In order to address the question to what degree WLE is associated with health-specific life expectancies, we first compare the four indicators in 2017 for 26 European countries. In most countries, women have lower levels of economic activity than men in both age groups, which is reflected in their lower WLE (Figs. 1 and 2). Exceptions are the Baltic States, Finland, and Bulgaria where women aged 50–59 show higher WLE, which remains true for women aged 60–69 in Estonia and Latvia. Overall, women aged 50–59 have on average seven remaining economically active years and women aged 60–69 have 2.3 years, whereas their male counterparts aged 50–59 can expect about eight remaining years and those aged 60–69 can expect about 3.2 years (Figs. 1 and 2). The maximum remaining life-years that are possible in each of the two age groups is on average 9.9 years between age 50–59 and 9.7 years between age 60–69 for women, and 9.7 years between age 50–59 and 9.3 years between age 60–69 for men. These number of years represent the maximum values that would theoretically be possible for each of the four indicators.

A striking observation for women and men aged 50–59 is that in all but six instances—the exceptions being women in Croatia, Cyprus, Greece, Italy, Malta, and Spain—the remaining WLE is larger than the expected remaining years in good health for at least one of the three health indicators. The number of years in good physical health is the largest of the three life expectancies in the majority of countries, for men as well as women, and good cognitive life expectancy in most instances the second largest one. The conventional calculation for healthy life years, based on the GALI question, reveals that the number of economically active years is larger than the number of healthy years in 19 out of 26 countries for women and in 22 out of 26 countries for men. The two health measures that are based on indicators that are having clear relevance for work ability and employability provide a less dire picture: for ages 50–59, years with good physical and cognitive health are in most instances larger than working life expectancy for women. For men, the picture is more mixed: WLE and both of these health indicators are around equal length (e.g. in Czechia, France, and Germany) or WLE ranges between the two health indicators with more often than not CLE being shorter and PLE being longer than WLE.

A very different picture presents itself when looking at persons aged 60–69, where WLE is almost universally significantly lower than the expected number of healthy years for any health measure (Fig. 2). In most countries, men and women can expect on average seven to eight years out of the possible ten years in good physical health, while they can expect only six to seven years with good cognitive functioning. When it comes to HLE, the average number of years without an activity limitation is 5.6 years for men and 5.3 years for women. This implies that HLE is lower than the number of remaining years in good health for the two other health life expectancies with the exception of Bulgarian, Cypriot, Greek, Italian, Maltese, Spanish, and Slovakian men. This overall pattern, that also appeared already for persons aged 50–59, might be due to the different nature of our health measures: HLE is based on self-reported information whereas the other two measures are performance measures. Another explanation might be that limitations that are picked up through the GALI measure are mostly not directly relevant for individual labour market participation and allow being economically active in spite of their presence.

### Trends in life expectancies, 2004–2017

**Fig. 3:**
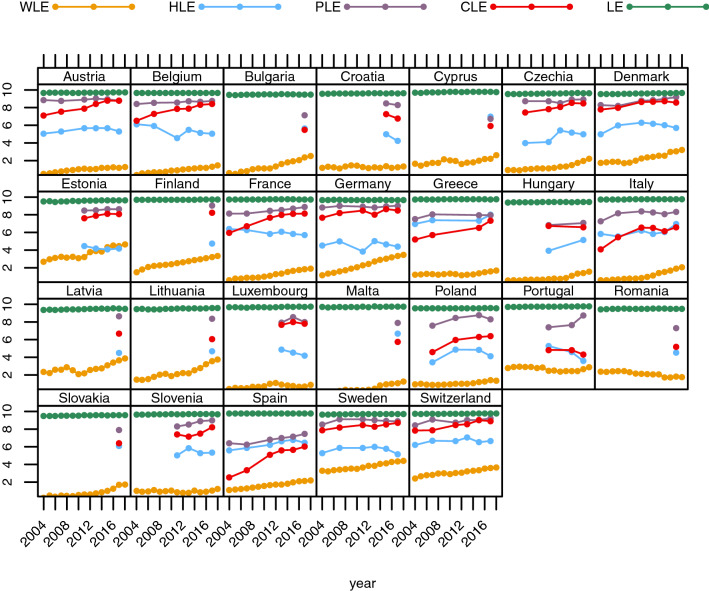
Trends in women’s partial life expectancies (WLE, HLE, PLE, CLE, and LE) between ages 60 and 69 from 2004 to 2017 for selected European countries

Our investigation of time trends of the different partial life expectancy indicators shows that WLE increased for women age 50–59 since 2004 in particular in countries starting at relatively low level such as Austria, Belgium, Greece, Italy, Luxembourg, Malta, Poland, Slovenia, and Spain (Fig. 7 Appendix). WLE in countries like Denmark, Estonia, Finland, and Sweden remained at a more or less constant level as women could expect about eight remaining years in the labour market already in 2004. Interestingly, women could not only expect many years in good physical health in 2017, this indicator has been at quite high levels during the previous decade and turns out to be the most stable over time of all four indicators. The number of remaining years with good cognitive functioning increased over time in most countries and approached the level of good physical functioning. Women’s remaining HLE showed no uniform pattern over time for the 26 countries, increasing for some countries, stagnating for a few, and decreasing for others. Women aged 60–69 show a similar pattern as their younger counterparts with WLE being the exception with a significantly lower number of expected economically active years (Fig. 3).

**Fig. 4:**
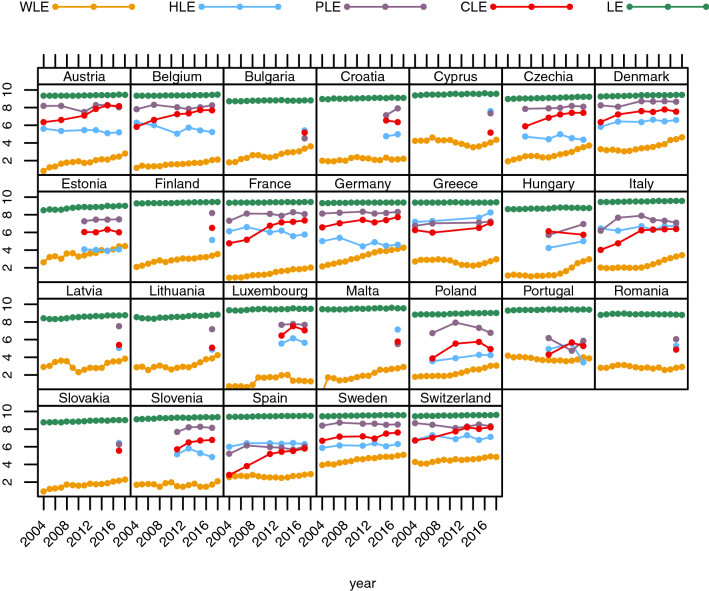
Trends in men’s partial life expectancies (WLE, HLE, PLE, CLE, and LE) between ages 60 and 69 from 2004 to 2017 for selected European countries

WLEs for men age 50 to 59 have all along been at much higher levels than those of women and therefore show only a minor increase over time with exception of Hungary, Poland, and Romania (Fig. 8 Appendix). However, between age 60 and 69 there is a clear increase in WLE until 2017 for countries like Austria, Bulgaria, Czechia, Finland, France, Germany, Italy, Hungary, Malta, Poland, and Slovakia, even when starting with two or fewer remaining economically active years in 2004 (Fig. 4). The trend of an increasing number of remaining years with good cognitive ability over the last decade for men in the older age group was similar to those observed among women of the same age (see Figs. 3 and 4). The magnitude of the gap between economically active years and healthy years declined almost universally over time for 60–69-year-old men. The trend of HLE does not provide a clear pattern for either age group, as HLEs of men increased in some countries and decreased or stayed about constant in others. Life expectancies (LE), which define the maximum number of years possible for each indicator, stayed constant for women in both age groups and men age 50–59 and increased only slightly for men age 60–69 (cf. Figs. 3, 4, 7 and 8). Since observed changes in the four other indicators that incorporate life table information were more pronounced than changes in LE, this implies that changes in the four other indicators are due to changes in underlying prevalences and not or much less to changes in mortality.

This diverse pattern of increases, decreases, and stagnation across the four partial life expectancy indicators is also reflected in the estimated correlations between WLE and the health-specific measures. Overall, life expectancies between age 60 and 69 show lower correlations than between age 50 and 59. As also indicated by inspection of the longitudinal trajectories, WLE and CLE between ages 50 and 59 have the highest correlation with 0.43 for men and 0.47 for women (Table 3). The fact that WLE and HLE show the lowest and partly insignificant correlations could be interpreted as HLE not being a very good indicator to measure work ability and employability.

**Table 3:** Correlation coefficients between WLE and HLE, PLE, and CLE between ages 50 and 59 and 60 and 69, by sex.

Correlation between	Women 50–59	Men 50–59	Women 60–69	Men 60–69
WLE and HLE	−0.37	0.23	−0.04^a^	0.11
WLE and PLE	0.22	0.20	0.30	0.14^a^
WLE and CLE	0.47	0.43	0.33	0.21

Pooled sample of our 26 selected countries and all available years, 2004 to 2017

^a^ indicates no significance. All other correlations are significant at least at the 5%-level

### Education-specific life expectancies

The trends investigated in the previous section showed that the magnitude of the gap between WLE and healthy years declined slightly over time as WLE has been increasing in many countries, but that there is still a considerable gap in most countries between the number of years spent working and the number of years in good health. However, reporting and comparing only country averages might disguise heterogeneities between different socio-economic groups when it comes to economic activity and health status (Cambois et al. 2020). Education-specific differences in the length of working lives as well as the number of years in respective health conditions are the results of the combination of differences in life expectancies and in health status by education. With both measures being positively correlated with educational attainment, persons with higher levels of education in general not only live longer but they also spend more years working and in good health. Availability of comparable data on life expectancy by highest level of educational attainment restricts this part of the analysis to 15 countries.

**Fig. 5:**
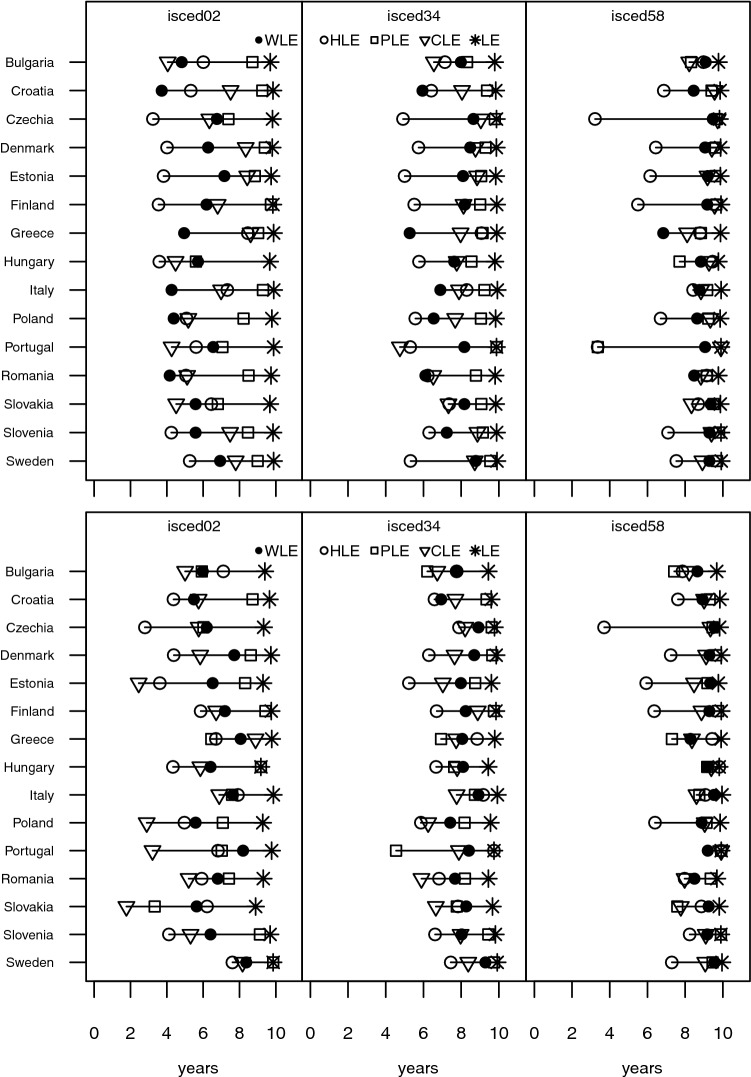
Partial life expectancies (WLE, HLE, PLE, CLE) of women (top) and men (bottom) between ages 50 and 59 for three educational groups for selected European countries in 2017. Note: at most lower secondary education (ISCED 0–2), upper secondary and post-secondary non-tertiary education (ISCED 3–4), and short-cycle tertiary education or higher (ISCED 5–8)

We find the expected educational gradient for working life expectancy as well as our three health indicators, but to varying degrees. Our results highlight that highly educated men and women aged 50 can expect to spend the great majority of the subsequent 10 years in good physical and cognitive health as well as economically active (see Fig. 5). This is less true for upper and post-secondary educated, where the number of economically active years is lower and the range of number of years in good health for the three health indicators increases. On average the magnitude of the gap between remaining years in good health (physical or cognitive) and economic activity peaks for those at most lower secondary educated. While working life expectancy between countries varies more for women than for men aged 50–59 (cf. Figure 1), it shows the smallest range of educational discrepancies (addressed by the deviation from the group average) next to PLE for both sexes (SD for WLE and PLE: 1.5 and 1.1 for women; 1.1 and 1.0 for men). CLE, on the other hand, shows the biggest educational discrepancies (SD: 1.7 for women; 2.1 for men).

**Fig. 6:**
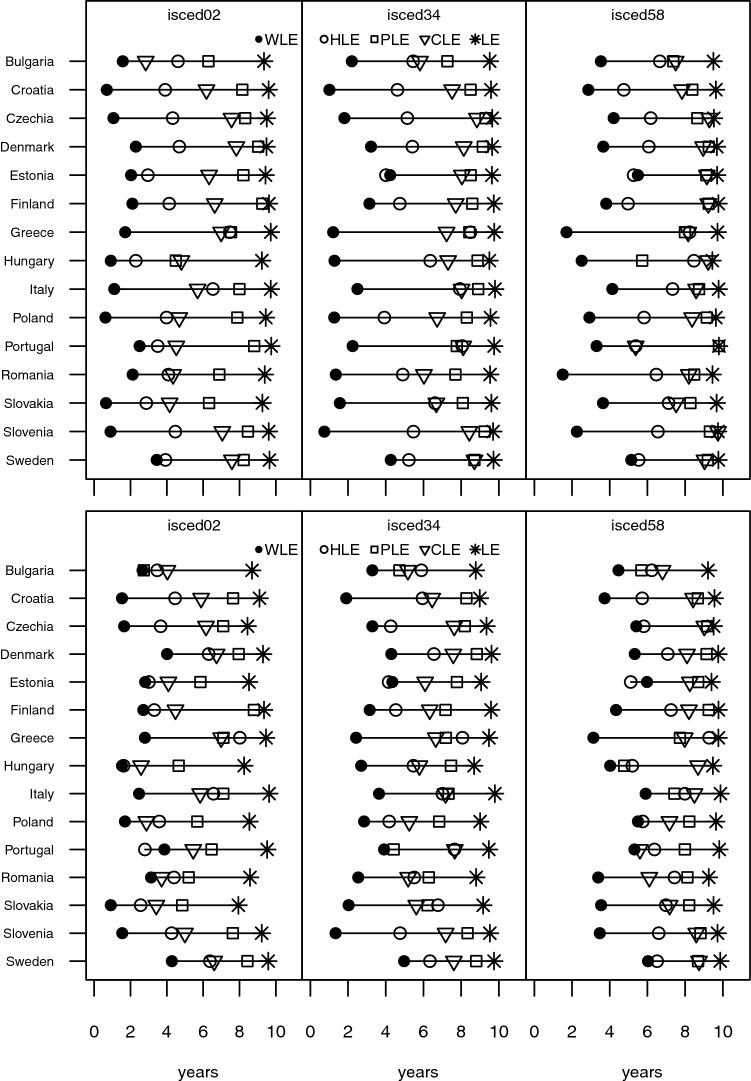
Partial life expectancies (WLE, HLE, PLE, CLE) of women (top) and men (bottom) between ages 60 and 69 for three educational groups for selected European countries in 2017. See note for Fig. 5

**Fig. 7:**
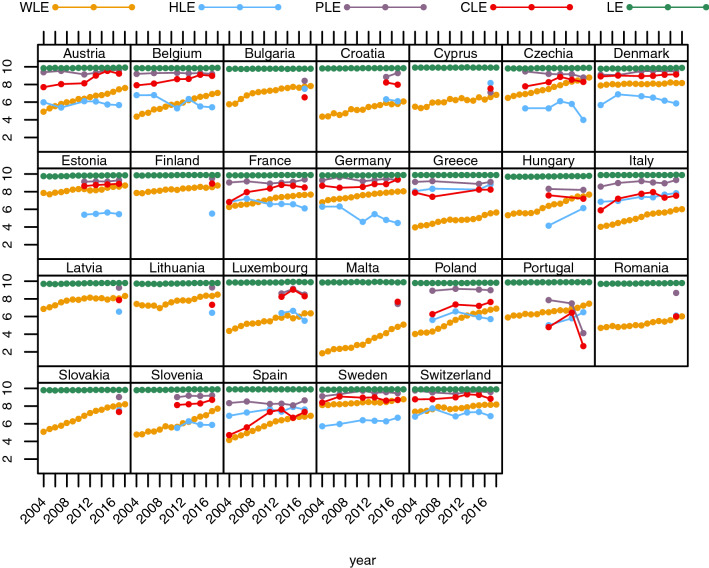
Trends in women’s partial life expectanies (WLE, HLE, PLE, CLE, and LE) between ages 50 and 59 from 2004 to 2017 for selected European countries

**Fig. 8:**
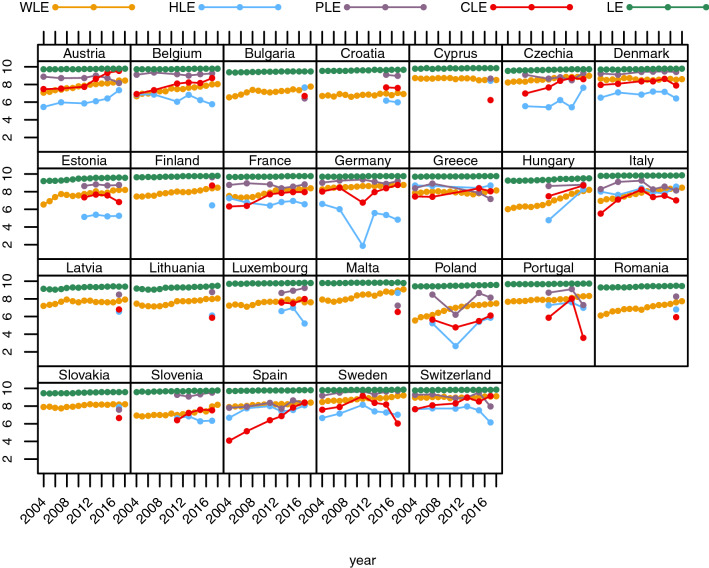
Trends in men’s partial life expectanies (WLE, HLE, PLE, CLE, and LE) between ages 50 and 59 from 2004 to 2017 for selected European countries

**Fig. 9:**
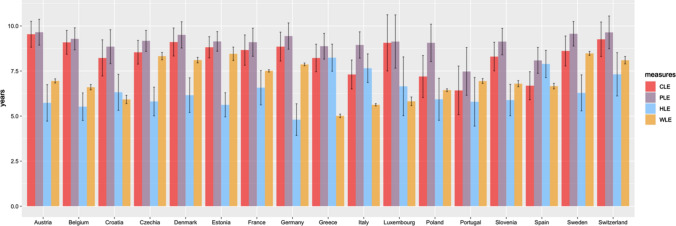
Partial life expectancies (WLE, HLE, PLE, and CLE) and 95% CIs between ages 50 and 59 for women in 2015

**Fig. 10:**
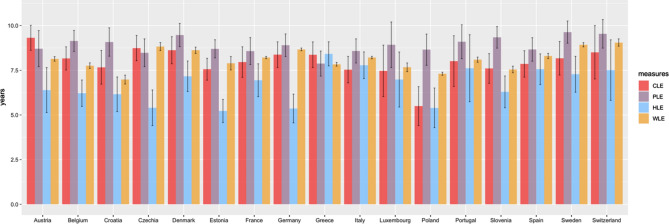
Partial life expectancies (WLE, HLE, PLE, and CLE) and 95% CIs between ages 50 and 59 for men in 2015

**Fig. 11:**
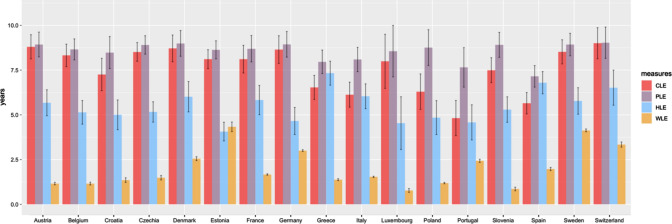
Partial life expectancies (WLE, HLE, PLE, and CLE) and 95% CIs between ages 60 and 69 for women in 2015

**Fig. 12:**
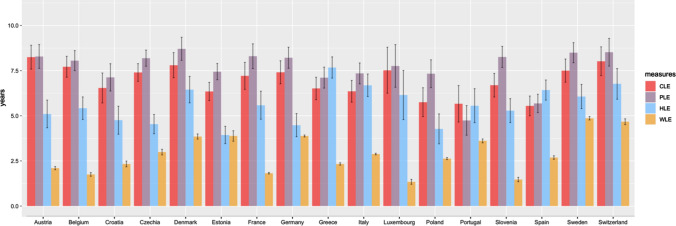
Partial life expectancies (WLE, HLE, PLE, and CLE) and 95% CIs between ages 60 and 69 for men in 2015

The general picture is similar for men and women between ages 60 and 69: the higher the education level, the more years in good health can be expected (see Fig. 6). However, the variance between the health indicators within education categories is significantly larger for the older age group than for members belonging to the younger age group. Particularly men aged 60 and at most lower secondary educated can expect fewer years of remaining economic activity than those with higher education, but they are also deprived when it comes to remaining years in good cognitive health or general health, implying that there is also much less leverage to increase the number of economically active years.

In general, the differences in life expectancy (LE) by education for persons aged 60–69 are noticeably larger for men than for women (see Fig. 6). This entails that the effect of differences in mortality between men with different education levels play a larger role for the values of the four partial life expectancy indicators than in the case of women. For the latter, observed education differences in the number of economically active and healthy years are mostly driven by differences in health prevalences.

## Discussion

When only considering current economic activity, the potential to increase working life is larger between ages 60 and 69 than between ages 50 and 59. This is nothing new and can be deducted from looking at participation rates alone. However, once we start to compare the number of years that someone can expect to be economically active with the number of years that he or she can expect to experience in good health, the picture becomes much more nuanced. Jagger et al. (2011) found large inequalities between European countries when it comes to health expectancies, and HLE at age 50—defined in various ways—varied significantly across countries, pointing towards the importance of country-specific characteristics in shaping the disablement process. Our results point in the same direction. However, whereas their definitions of health focused on aspects of disablement, our focus in this study was on health aspects that are related to employability, namely PLE and CLE. For the purpose of comparability with other health research, we compared WLE to the common health measure of HLE. In countries where female LFP is relatively high, 50–59-year-old women can currently expect more years of economic activity than in good health based on HLE, while the number of years expected in good health based on our measures of cognition and physical strength are mostly larger than WLE. The results for men are similar but since male labour force participation is higher in general, 50–59-year-old men in more countries than women have already now WLEs that are larger than HLE and even CLE. This implies more room to increase the number of active years between age 50 and 60 for women—keeping in mind noticeable differences across countries when it comes to the number of years in good health.

For 60–69-year-old women and men, WLE is still significantly lower than the expected number of years in good health according to our health measures, but also for this age group, the size of this potential to increase working lives varies greatly across countries. Looking at correlations between WLE and the three health indicators, CLE and WLE show the largest and most consistent correlation for women as well as men for expectancies for both age groups we analysed.

Retired and employed persons above age 65 differ with respect to their type of (previous) occupation, working conditions, and health status, providing evidence that increasing the length of working life will be harder on some population groups than others (Wahrendorf et al. 2017). As our results demonstrate, there are large heterogeneities within each indicator: socio-economic status is a strong predictor at the individual level of health status, and these differences are reflected in the education-specific calculations of our four indicators. This confirms previous findings, for example for France (Cambois et al. 2011) and for Germany (Hasselhorn and Rauch 2013). Working life expectancy shows a clear education gradient for individuals in both age groups. However, this gradient also exists for each of the three health indicators. This implies that the leverage to increase the number of economic active years that seems to be there across the board when comparing simple averages of the four indicators is actually distributed unevenly across individuals with different education levels. While there is hardly any room to increase WLE for men and women between ages 50 and 59 for the highest education group, since it is already in most countries close to 10 years, there is still room for those with less than tertiary education, even when considering the health dimension. For ages 60–69, a different picture presents itself: in spite of WLE being higher for men and women with upper secondary education or higher than for the lowest education group, the leverage to increase WLE further seems also higher for the two higher education groups due to the simultaneously larger expected number of years in good health.

Overall, PLEs are larger than any of the other three indicators, an observation that holds true for both age groups and sexes. While this is good news, it is clear from current and expected future job demands and the literature that physical strength—while still necessary for certain occupations and tasks—will be less important for a lot of occupations compared to cognitive abilities. These skills play already nowadays a pivotal role for employability and productive employment at older ages and will likely be even more important in the future (World Economic Forum 2018). Thus, it will be crucial to maintain high levels of cognitive functioning well beyond age 50 in order to match labour demand. Our finding that the number of years with good cognitive functioning increased in the majority of countries between 2004 and 2017 for persons aged between 60 and 69 is a positive development and might partly be due to shifts in the educational attainment structure towards higher education levels within this age group.

The presented results for three health indicators provide a snap-shot of the current situation of older adults in the countries that were part of our analysis. It should be clear that the observed prevalences and resulting health expectancies are not only the result of living and working conditions of persons above age 50 but that they are shaped by individuals’ experiences over their entire life courses. If the goal is to increase the number of years that older adults enjoy in good health—for their own good, for keeping health-related costs in check and also against the background of further extensions of working life—a holistic approach that considers many aspects over the life course is warranted (Eurofound 2015). These aspects concern characteristics and circumstances of the individual as well as characteristics of the job (Oltmanns et al. 2017).

What do our results imply for the future? Due to educational expansions in the past, future older adults will be on average better educated than current ones (Loichinger 2015). Those who are now 30–49 years old will during the next 2 decades replace those who are currently 50–69 years old. If educational differences in health status prevailed at around the presently observed levels, this replacement could entail on average healthier older adults and workers, with the ability to work until higher ages. Still, given foreseeable persistent differences in health status of persons with varying socio-economic status, adequate solutions will have to be found for those whose health does not keep up with further increases in statutory retirement ages. Considerations to prolong working lives within each country should be accompanied by a thorough analysis of factors, including health, that play a role for the ability to continue working in the given context, as it was for example done by the Dutch Ministry of Social Affairs and Employment (van der Mark-Reeuwijk et al. 2019).

The narrative for the need to extend working lives in order to react to increasing longevity and fears of labour shortages is prevalent at national levels as well as on the EU level. While this narrative itself should come under more scrutiny in the context of each country’s individual demographic and economic circumstances, there are several issues when it comes to longer working lives that to date are not being adequately dealt with. These include but are not limited to issues concerning expected increases in social inequalities, developments of the demand for older workers and the quality of future jobs, and the provision of life-long education opportunities (Phillipson 2019).

## Limitations

Estimating health expectancies based on transition rates and the multistate life table method provides exact point estimates of the true period value. The method allows the explicit inclusion of disability incidence and recovery. The main drawback is the requirement of longitudinal data for health. As has been shown by Mathers and Robine (1997), results for disability-free life expectancy calculated with the multistate life table method and Sullivan’s method, respectively, showed very little differences in the results as long as changes in disability prevalence happen smoothly and relatively regular. This makes Sullivan’s method suitable to monitor health developments in the long term and at the population level. Changes in our three health indicators—i.e. the age-specific developments of their prevalences—happen gradually and without abrupt jumps or drops. Discrepancies of a smooth trend are only found for men aged 50–59 in Germany, Poland, and Portugal as well as for women aged 50–59 in Portugal, which are likely due to very small sample sizes in the relevant age groups.

The situation is trickier when it comes to using Sullivan’s method to calculate working life expectancies; the financial crisis years (2007–2012) constituted a substantial effect on labour market outcomes. As Dudel et al. (2018) demonstrated for Spain, estimates of WLE based on the Sullivan’s method can lead to biased results in cases where labour force participation rates are changing abruptly. However, the effect seems to be foremost the slower adjustment to new levels of WLE after an exogenous shock for calculations with the Sullivan method compared to Markow chain calculations. After a few years, the values were at similar levels again. Since the situation in Spain was more extreme than in other European countries and since we are using labour force participation rates (i.e. a combination of the employed and the unemployed) and focus on long-term trends, we expect that this approach is still valid for our purposes (Lozano and Rentería 2019). Still, this limitation has to be kept in mind when interpreting the results for WLE in this paper, particularly developments over time.

A general aspect why the multistate method is superior to Sullivan’s method for calculations of working life expectancies is the fact that it allows for transitions between different labour market states (e.g. employment, unemployment, sick leave, early retirement). This possible consideration of more detailed information on labour market statuses also allows for the estimation of the amount of time spent in these different states, not just the economically active time. Such detailed analyses are usually performed for single countries and not in a comparative way, due to the required nature of the data (longitudinal samples, detailed breakdown into different statuses).

In this study, we use objective and subjective health measures, which might be attended by health misperception (over- and underestimation). Our objective health indicators are already at a high level; thus, a correction of the potential underestimation would result in even higher levels. We might suspect overestimation in our subjective measure due to social norms or cultural differences in reporting, but several studies verified the validity and reliability of GALI (Van Oyen et al. 2018). Further, we acknowledge practice effects in repeated cognitive tests (Hale et al. 2020; Lièvre et al. 2008; Mazzonna and Peracchi 2012). However, literature shows that practice effects occurred mostly between first and second testing (Alexander et al. 2003; Lièvre et al. 2008) and that these effects are greater at the lowest performance level (the lowest quartile) (Gross et al. 2015). In this study, we investigate a rather demanding cognitive level (recalling at least 5 out of 10 words). Moreover, we also attribute increases in cognitive performance by cohorts to the well-investigated Flynn effect, which was also shown for only first time participants with SHARE data (Weber et al. 2017).

## Conclusions

In this paper, we took a macro-level approach and compared developments of working life expectancies and health expectancies for health dimensions that are relevant for older adults’ work ability. While we found large differences across countries, overall, there is potential to increase the expected number of economically active years for men as well as women between ages 60 and 69. However, our education-specific analyses revealed large differences between socioeconomic subgroups of the population when it comes to the size of this potential. This heterogeneity between education groups when it comes to health and their ability to work beyond currently observed labour market exit ages has to be taken into account when working lives are being extended.

### Electronic supplementary material

Below is the link to the electronic supplementary material.

Supplementary file 1 (CVS 15kb)

## Appendix A: Additional Figures

See Figs. 7, 8, 9, 10, 11 and 12.
